# The role of the dorsal raphé nucleus in reward-seeking behavior

**DOI:** 10.3389/fnint.2013.00060

**Published:** 2013-08-27

**Authors:** Kae Nakamura

**Affiliations:** ^1^Department of Physiology, Kansai Medical UniversityHirakata, Japan; ^2^Precursory Research for Embryonic Science and Technology, Japan Science and Technology AgencyKawaguchi, Japan

**Keywords:** 5-HT, dopamine, raphé, saccade, primate, reinforcement, reward

## Abstract

Pharmacological experiments have shown that the modulation of brain serotonin levels has a strong impact on value-based decision making. Anatomical and physiological evidence also revealed that the dorsal raphé nucleus (DRN), a major source of serotonin, and the dopamine system receive common inputs from brain regions associated with appetitive and aversive information processing. The serotonin and dopamine systems also have reciprocal functional influences on each other. However, the specific mechanism by which serotonin affects value-based decision making is not clear. To understand the information carried by the DRN for reward-seeking behavior, we measured single neuron activity in the primate DRN during the performance of saccade tasks to obtain different amounts of a reward. We found that DRN neuronal activity was characterized by tonic modulation that was altered by the expected and received reward value. Consistent reward-dependent modulation across different task periods suggested that DRN activity kept track of the reward value throughout a trial. The DRN was also characterized by modulation of its activity in the opposite direction by different neuronal subgroups, one firing strongly for the prediction and receipt of large rewards, with the other firing strongly for small rewards. Conversely, putative dopamine neurons showed positive phasic responses to reward-indicating cues and the receipt of an unexpected reward amount, which supports the reward prediction error signal hypothesis of dopamine. I suggest that the tonic reward monitoring signal of the DRN, possibly together with its interaction with the dopamine system, reports a continuous level of motivation throughout the performance of a task. Such a signal may provide “reward context” information to the targets of DRN projections, where it may be integrated further with incoming motivationally salient information.

## Introduction

Serotonin (5-hydroxytryptamine, 5-HT) is present in almost all organisms from plants to vertebrates. In mammals, 5-HT has been found in all organs, such as the brain, gut, lung, liver, kidney, and skin, as well as platelets. Such a wide distribution indicates that 5-HT is an essential chemical for all living animals. In the brain, the distribution of 5-HT projections is widespread, regulating the activity of almost all brain regions. Thus, it is no surprise that 5-HT has been implicated in a variety of brain functions, such as the sleep-wake cycle, appetite, locomotion, emotion, hormonal regulation, and as a trophic factor.

In addition to the “basic” brain functions described above, the role of 5-HT in cognitive functions, including attention, control of impulsivity, coping with stress, social behavior, value-based decision making, and learning and memory, has also captured a great deal of attention. The breakdown of the 5-HT system is often associated with neuropsychiatric diseases including depression, schizophrenia, drug abuse, autism, and Parkinson's disease. However, the specific mechanisms by which 5-HT is involved in these cognitive processes are not yet clear.

Among the possible functions of 5-HT, this review will focus on its role in reward-seeking behavior. There are already good reviews about the role of 5-HT in value-based decision making, often being compared with dopamine function. For example, it has been proposed that the tonic and phasic dopamine and 5-HT systems represent value and action, which are not independent, in an opposite manner. Thus, dopamine may be involved in behavioral activation to obtain rewards and 5-HT may be involved in inhibition in the face of punishment (Boureau and Dayan, 2010; Cools et al., 2011). This unified model can account for the variety of aspects of decision making, including response vigor, time discounting, switching, and risk sensitivity, observed in behavioral-pharmacological experiments in animals and humans. The aim of this review is to further focus on the anatomical and physiological evidence of the 5-HT system and link it with the above findings. I will first review the anatomical evidence that supports the involvement of the raphé nuclei, the origin of 5-HT, in reward-dependent behavior. Among the raphé nuclei, I will focus on the dorsal raphé nucleus (DRN) because it has strong anatomical and physiological connections with the brain areas that are related to reward processing. Second, I will introduce pharmacological studies that examined the impact of changes in the brain levels of 5-HT on reward-seeking behavior. Although the results are mixed, depending on the affected brain regions and the type of 5-HT receptors examined, these studies generally support the inhibitory effect of 5-HT on reward-seeking behavior.

The behavioral pharmacological studies examined how 5-HT is utilized at the projection targets. On the other hand, it is also critical to reveal when and in which situations 5-HT is secreted or when DRN neurons are activated in real time. Recently, several research groups measured the activity of single DRN neurons while animals performed behavioral tasks. I will review the results of single unit recordings from the DRN, including our recent experiments in monkeys. The results show that DRN neuronal activity continuously keeps track of the expected and received reward value throughout the trials.

Finally, I will discuss the possible mechanisms by which 5-HT modulates value-based decision making, together with dopamine and other brain structures, such as the lateral habenula, amygdala, frontal cortex, and basal ganglia.

## Anatomical implication of the role of 5-HT in motivational behavior

There is a great amount of evidence demonstrating tight anatomical connections between the raphé nuclei and the brain areas that are related to reward (Azmitia and Gannon, 1986; Molliver, 1987; Jacobs and Azmitia, 1992; Michelsen et al., 2007).

Among the 9 raphé nuclei B1–B9 (Dahlstroem and Fuxe, 1964), those that are often discussed in relation to reward-related behavior are the DRN, which is the largest group (B7), lumped together with B6, and the median raphé nucleus (MRN), which consists of B8 and B5.

### Input to the DRN that may be involved in reward processing (Figure 1, left)

The DRN receives projections from many brain areas that have been associated with reward and punishment. These areas tend to project to distinct divisions of the DRN (Aghajanian and Wang, 1977; Sakai et al., 1977; Behzadi et al., 1990; Peyron et al., 1998).

Cortical areas projecting to the DRN include the medial prefrontal (Arnsten and Goldman-Rakic, 1984), lateral and medial orbital, cingulate, infralimbic, and insular cortices (Arnsten and Goldman-Rakic, 1984; Sesack et al., 1989; Amat et al., 2005). At least a part of the projection from the medial frontal cortex is via GABA interneurons in the raphé nuclei (Arnsten and Goldman-Rakic, 1984; Hajos et al., 1998; Varga et al., 2001, 2003; Jankowski and Sesack, 2004), which in turn project to 5-HT neurons.

Subcortical areas projecting to the DRN include the amygdala (Peyron et al., 1998; Lee et al., 2007), substantia nigra pars reticulata (SNr), ventral pallidum, preoptic area, claustrum, bed nucleus of the stria terminalis, zona incerta, medial and lateral preoptic areas, hypothalamus, and, most prominently, the lateral habenula nucleus (Pasquier et al., 1976; Aghajanian and Wang, 1977; Wang and Aghajanian, 1977c; Herkenham and Nauta, 1979; Stern et al., 1979; Kalen et al., 1989; Peyron et al., 1998; Varga et al., 2003), whose projection is through the fasciculus retroflexus. The lateral habenula is a brain region that represents negative motivational values, such as reward omission and aversive stimuli (Matsumoto and Hikosaka, 2007, 2009; Hong and Hikosaka, 2008) and transmits these signals to midbrain dopamine neurons and the DRN. Many studies have reported an inhibitory effect from the habenula to the DRN via the rostromedial tegmental nucleus (RMTg). Stimulation of the habenula suppresses the activity of DRN 5-HT neurons (Aghajanian and Wang, 1977; Wang and Aghajanian, 1977c; Stern et al., 1979; Nishikawa and Scatton, 1984, 1985, 1986; Scatton et al., 1984; Nishikawa et al., 1986; Ferraro et al., 1997; Varga et al., 2003) and decreases 5-HT release in the caudate nucleus and substantia nigra (Reisine et al., 1982; but see Kalen et al., 1989).

The hypothalamus is also an important source of reward information for the DRN (Celada et al., 2002). Hypothalamic orexin neurons are activated by arousal, feeding, and rewarding stimuli (Mieda and Yanagisawa, 2002; Lee et al., 2005; Harris and Aston-Jones, 2006) and facilitate 5-HT release (Tao et al., 2006). The amygdala, in which neurons encode positive or negative motivational values (Ledoux, 2000; Belova et al., 2008), also sends projections to the DRN.

The dopamine neurons in the ventral tegmental area (VTA) and substantia nigra pars compacta (SNc) also project to the DRN and MRN (Kalen et al., 1988; Mansour et al., 1990; Peyron et al., 1995; Kitahama et al., 2000), which may exert facilitatory effects on putative 5-HT neurons in the DRN by D2-like dopamine receptor activation (Ferre and Artigas, 1993; Mendlin et al., 1999; Haj-Dahmane, 2001).

Finally, the activity of neurons in the raphé nuclei is regulated by 5-HT via the 5-HT1A receptor found on the somata and dendrites of neurons in the raphé nuclei, where it functions as a somato-dendritic auto-receptor (Wang and Aghajanian, 1977a; Gozlan et al., 1983; Verge et al., 1985; Carey et al., 2004).

**Figure 1:**
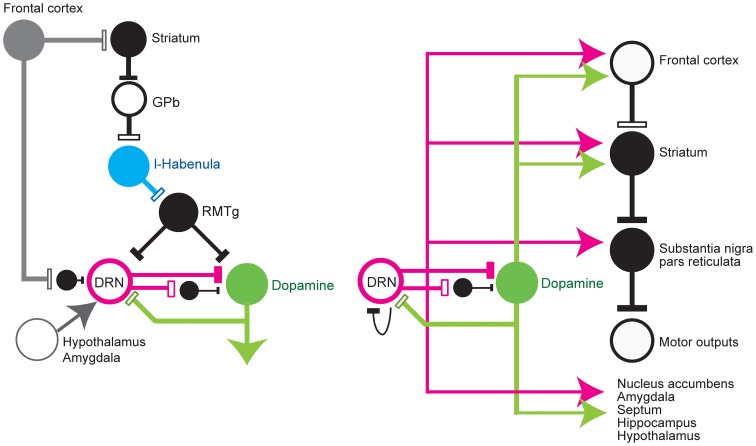
**Simplified diagram of input to (left) and output from (right) the dorsal raphé nucleus.** DRN, dorsal raphé nucleus; Dopamine, dopamine neurons; l-Habenula, lateral habenula; RMTg, rostromedial tegmental nucleus; and GPb, globus pallidus external and internal border (anatomically called the internal medullary lamina). The open and filled rectangles correspond to excitatory and inhibitory connections, respectively. The arrows indicate that the effect is unknown or excitatory and inhibitory effects have been reported.

### Output from the raphé nuclei (Figure 1, right)

Efferent projections from the raphé nuclei are widespread, but constitute a topographic organization along the rostrocaudal and medial-lateral axes (Imai et al., 1986; Abrams et al., 2004; Lee et al., 2008). Separate ascending pathways have been described in rats and primates. In the rat, the largest pathway is the medial forebrain bundle, which carries fibers from the MRN and DRN to a wide range of target areas in the forebrain. In primates, a significant number of these fibers (~25%) are heavily myelinated (Azmitia and Gannon, 1986), and the largest pathway appears to be the dorsal raphé-cortical tract, which enters the cortex through the internal capsule (Azmitia and Segal, 1978; Azmitia and Gannon, 1986). Many projection sites include areas that are associated with reward processing, such as the neocortex, nuclei in the basal ganglia, nucleus accumbens, amygdala, septum, hippocampus, and hypothalamus (Azmitia and Segal, 1978; Azmitia and Gannon, 1986; Molliver, 1987; Vertes, 1991; Peyron et al., 1998).

The innervations from the DRN have several characteristics. First, individual DRN neurons give rise to several sets of collateral (branched) projections to distinct, but functionally related, targets. Single DRN 5-HT neurons project to the septum and the entorhinal area, both of which are essential for normal hippocampal function (Kohler et al., 1982), to various combinations of the olfactory cortex, septum, and medial thalamus (De Olmos and Heimer, 1980), to the prefrontal cortex and nucleus accumbens (Van Bockstaele et al., 1993), and to the central nucleus of the amygdala and paraventricular nucleus in the hypothalamus, both of which are involved in central autonomic control, anxiety, and conditional fear (Petrov et al., 1994; Lowry, 2002). This branching is also observed in the DRN projections to the sensory-motor areas, such as the lateral geniculate body and superior colliculus (Villar et al., 1988), which are important for visual information processing, and the substantia nigra subthalamic nucleus and caudate-putamen (Van Der Kooy and Hattori, 1980; Imai et al., 1986), which are involved in the execution of movement. These serotonergic collateral projections to functionally and anatomically related targets could facilitate the integrated and temporally coordinated modulation of multiple brain regions.

Second, 5-HT acts on all major dopaminergic pathways, i.e., nigrostriatal, mesocortical, mesolimbic, and tuberoinfundibular. The interaction of the 5-HT system with the dopamine system has been documented in the frontal cortex and basal ganglia nuclei, which form part of the nigrostriatal, mesocortical, and mesolimbic dopamine pathways. The fourth dopamine pathway, the tuberoinfundibular pathway, projects from the arcuate nucleus to the median eminence in the hypothalamus. Here, dopamine inhibits the secretion of prolactin from the anterior pituitary gland during the resting state. It is also known that stressful events that evoke prolactin release seem to rely, at least partially, on central serotonin function (Bregonzio et al., 1998). In terms of receptor types, although with some exceptions, receptor types such as 5-HT1A, 5-HT1B, 5-HT2A, 5-HT3, and 5-HT4 facilitate dopamine release, while 5-HT2C exerts tonic inhibition on dopamine release (for review, Alex and Pehek, 2007).

Here, in the first section, we discuss primarily the anatomical connections of the DRN to the reward-related brain areas. In the second section, we will focus more on the differential functional effects of 5-HT.

#### Projections to the striatum and SNr

Among the widespread efferent projections of the DRN, those to the basal ganglia structures, especially the striatum and substantia nigra, may be particularly important for the control of the reward-dependent modulation of action (monkey, Lavoie and Parent, 1990; rat Van Der Kooy and Hattori, 1980; Imai et al., 1986). In monkeys (Lavoie and Parent, 1990; Haber, 2003), 5-HT terminals are particularly abundant in the ventral striatum, including the nucleus accumbens, ventrolateral region of the putamen, and ventromedial region of the caudate nucleus. The influence of 5-HT depends on the type and location of its receptors. High levels of 5-HT1B, 2A, and 2C receptors are reported in the striatum (Wright et al., 1995; Eberle-Wang et al., 1996).

Many reports examining the function of 5-HT in the striatum have focused on its effect on dopamine release. Electrical stimulation of the DRN enhanced dopamine release in the nucleus accumbens, but reduced it in the dorsal striatum; however, the specific effect depends on the type of receptors present. The facilitatory effect of endogenous 5-HT on dopamine release in the nucleus accumbens depends on the presence of 5-HT2A and 5-HT3 receptors, and not on 2B/2C receptors. Conversely, 5-HT2C receptors tonically inhibit dopamine release in the dorsal and ventral striatum (Jiang et al., 1990; Chen et al., 1991; De Deurwaerdere et al., 1998). The activation of 5-HT1B in the nucleus accumbens reportedly attenuated dopamine-dependent responses to a conditioned reward (Fletcher and Korth, 1999; but see Galloway et al., 1993).

The SNr is one of the major targets of the DRN in rats (Dray et al., 1976; Fibiger and Miller, 1977; Azmitia and Segal, 1978; Van Der Kooy and Hattori, 1980; Wirtshafter et al., 1987; Corvaja et al., 1993; Van Bockstaele et al., 1994; Moukhles et al., 1997), cats (Mori et al., 1987), and monkeys (Lavoie and Parent, 1990). In monkeys, 5-HT innervations are particularly dense in the SNr, but much less so in the SNc (Lavoie and Parent, 1990). Coexpression of 5-HT2C receptor mRNA with glutamic acid decarboxylase, but not with tyrosine hydroxylase mRNA, indicates that 5-HT2C receptors are restricted to GABAergic neurons (Eberle-Wang et al., 1997). The functional significance of 5-HT in the SNr, however, is not well understood.

#### Projections to the SNc and VTA

Electron microscopy studies have shown that 5-HT neurons make direct synaptic contacts with dopaminergic and non-dopaminergic neurons in the VTA (Herve et al., 1987; Van Bockstaele et al., 1994), indicating the direct and indirect influence of the raphé nuclei on the midbrain dopamine system. Electrical stimulation of the MRN (Dray et al., 1976) and DRN (Trent and Tepper, 1991; Gervais and Rouillard, 2000) inhibits the majority of (but not all) the activity of dopamine neurons. Further studies showed that the effect of 5-HT on midbrain dopamine neurons depends on the subtypes of 5-HT receptors present and the location of the dopamine neurons (Alex and Pehek, 2007). The systemic application of a 5-HT2C agonist decreased the baseline activity of dopamine neurons in a dose-dependent manner (Di Giovanni et al., 2000; Gobert et al., 2000), while the application of a 5-HT2C/2B antagonist caused a dose-dependent increase in the baseline and burst activity of dopamine neurons (Ugedo et al., 1989; Di Giovanni et al., 1999). As 5-HT2C receptors are mainly localized in GABAergic neurons in the SNr and VTA, which in turn inhibit dopamine neurons, the inhibitory effect of a 5-HT2C agonist on dopamine function is, at least in part, due to the GABA-mediated tonic inhibitory effect of 5-HT on mesolimbic and nigrostriatal dopamine function. On the other hand, the activation of VTA 5-HT1B receptors increases mesolimbic dopamine release, probably by inhibiting GABA release (Yan and Yan, 2001; Yan et al., 2004). Some authors have reported direct facilitatory effects of 5-HT on dopamine neurons *in vitro* (Nedergaard et al., 1988). In addition, 5-HT receptors located presynaptically on dopamine terminals or postsynaptically in dopamine projection areas could activate feedback loops, such as the striato-nigral, nucleus accumbens-VTA, or frontal-VTA pathways, thus indirectly altering the excitability of dopamine neurons in the SNc or VTA, resulting in changes in their baseline firing rates (Di Giovanni et al., 2010).

#### Projections to the amygdala

Several nuclei of the amygdala receive rich serotonergic innervations (Steinbusch, 1981). In rats, the rostral and medial subregions are dense projection sites of 5-HT neurons. In monkeys, 5-HT projections are found widely in the amygdala, with the highest concentration in the lateral division of the central nucleus and lateral-dorsal part of the bed nucleus of the stria terminalis (Sadikot and Parent, 1990; Freedman and Shi, 2001). The effect of DRN on neurons in the amygdala is reportedly inhibitory and mediated by direct DRN-amygdala serotonergic projections (Wang and Aghajanian, 1977b).

#### Projections to the hypothalamus

The hypothalamus plays a significant role in the processing of natural rewards, such as food and sex (Harris et al., 2005; Muschamp et al., 2007), and it receives strong inputs from the DRN (Nambu et al., 1999). Extracellular 5-HT levels increased in the medial and lateral hypothalamus during the anticipation and intake of food, but not after its consumption (Schwartz et al., 1990). Interestingly, this finding was interpreted in line with the reward-inhibiting and satiety-facilitating functions of 5-HT in the hypothalamus (Hoebel et al., 1989).

#### Projections to the cortex

The DRN also projects to virtually all cortical areas, and its effect can be excitatory and inhibitory, depending on which layers it projects to and the presence of different receptor types. Electrical stimulation of the DRN and MRN inhibits the majority of medial prefrontal cortex neurons via 5-HT1A (Hajos et al., 2003; Puig et al., 2005) or 5-HT2 (Mantz et al., 1990) receptors. Among several receptor types, 5-HT2A receptors are particularly dense in the prefrontal and anterior cingulate cortices (Pazos et al., 1985), and they are primarily located on the apical dendrites of pyramidal neurons (Jakab and Goldman-Rakic, 1998; Cornea-Hebert et al., 1999). Prefrontal 5-HT2A receptors may activate cortico-tegmental projection neurons, which in turn facilitate VTA dopamine neurons (Pehek et al., 2006). On the other hand, 5-HT2A/2C receptors are also present in the GABAergic interneurons of the cortex and may regulate glutamatergic output (Abi-Saab et al., 1999). 5-HT2C activation in the medial frontal cortex suppresses cocaine-seeking behavior (Pentkowski et al., 2010).

## 5-HT and the reward circuit

5-HT has long been implicated in a wide variety of motivational process; however, contrasting effects have been reported, many indicate a positive reward effect, but some others indicate a negative effect.

The positive reward effects of 5-HT have been described mainly in relation to brain self-stimulation experiments where animals perform operant responses such as pressing a bar to receive electrical stimulation of the brain. The majority of self-stimulation studies have focused on the medial forebrain bundle, which contains ascending dopaminergic fibers; however, several studies have also shown that stimulation of the raphé nuclei and their vicinity is equally effective (Miliaressis et al., 1975; Miliaressis, 1977; Rompre and Miliaressis, 1985). In addition, some pharmacological experiments using the systemic reduction of 5-HT reported attenuated cocaine-seeking behavior (Tran-Nguyen et al., 1999, 2001).

However, many lines of evidence indicate the inhibitory effects of 5-HT on the reward circuitry. The systemic injection of the 5-HT releaser *d*-fenfluramine (Fletcher, 1995) and the injection of 5-HT into the accumbens (Fletcher, 1996; Fletcher and Korth, 1999) attenuated conditioned responses to obtain amphetamine. The systemic reduction of 5-HT also reportedly enhanced reward-related behavior (Leccese and Lyness, 1984; Tran-Nguyen et al., 2001), while the findings of others depended on the type of reinforcement and the method used to reduce the function of 5-HT.

Experiments with the local injection of 5-HT inhibitors in the raphé nuclei support an inhibitory role of the raphé nuclei in motivational behavior. The local injection of a low dose of the 5-HT1A agonist 8-hydroxy-2-(di-n-propylamino)tetralin (8-OH-DPAT), which selectively inhibits serotonergic neurons in the MRN or DRN (Fletcher et al., 1993, 1995), and muscimol (Liu and Ikemoto, 2007) into the MRN induces conditioned place preference. It is of particular interest that these effects were reversed when the dopamine antagonists were administered systematically (Fletcher et al., 1999; Liu and Ikemoto, 2007) or directly into the nucleus accumbens (Muscat et al., 1989) or striatum (Fletcher and Davies, 1990; Fletcher, 1991), indicating that the reward effect of 5-HT antagonists may depend, at least partly, on the removal of the inhibitory influence of 5-HT on the mesolimbic dopamine system. Indeed, the systemic administration of 8-OH-DPAT increased the firing rate of the majority (75%) of dopamine cells studied and stimulated their bursting activity (Prisco et al., 1994).

The role of 5-HT in reward is complicated by the fact that it binds to a large number of receptor types that have different effects on reward-oriented behavior (Higgins and Fletcher, 2003). One of the principal receptor types involved in reward-oriented behavior may be the 5-HT2C receptor. This receptor's mRNA is expressed in the anterior olfactory nucleus, olfactory tubercle, claustrum, piriform and entorhinal cortices, lateral septal nucleus, amygdala, subiculum and ventral part of CA3, lateral habenula, subthalamic nucleus, SNr, VTA (Molineaux et al., 1989; Pompeiano et al., 1994; Wright et al., 1995; Eberle-Wang et al., 1997; Clemett et al., 2000), and dorsal and ventral (including nucleus accumbens) striatum, all of which are important parts of the reward-related circuitry. Another functional characteristic of the 5-HT2C receptor is that it possesses a high level of constitutive activity, even in the absence of agonist stimulation (Berg et al., 2008). It has been reported that neurons with 5-HT2C receptors in the nucleus accumbens and striatum are probably GABAergic projection neurons (Eberle-Wang et al., 1997). It was also suggested that all 5-HT2C mRNA-containing cells in the SNr and VTA are GABAergic, not dopaminergic, neurons. Thus, the tonic suppressive influence of 5-HT on dopamine neurons would be by 5-HT2C receptors acting on GABAergic neurons, which in turn suppress dopaminergic neurons in the VTA. This mechanism would allow 5-HT2C to exert a tonic influence on the activity of the mesocortical and mesolimbic dopaminergic pathways. Note, however, that a recent study provided anatomical and behavioral support for the localization of 5-HT2C receptors on dopamine neurons in the VTA (Ji et al., 2006). Altogether, 5-HT2C receptors tonically regulate, mainly by inhibition, dopamine release from the terminal regions of the nigrostriatal and mesolimbic pathways (Di Giovanni et al., 1999; Gobert et al., 2000).

As described above, many behavioral-pharmacological studies have reported the effects of 5-HT on the reward circuitry. However, the direction (positive or negative) of its effects should be analyzed carefully because it may vary depending on the method used to modulate 5-HT levels (e.g., systemic or local), the location of self-stimulation (Ahn et al., 2005), or the kinds of behavioral test used (Mosher et al., 2005; Hayes et al., 2009).

Another hypothesis for the role of the 5-HT system in reward-seeking behavior is that 5-HT regulates the timescale of reward prediction, such as the balance between immediate and delayed rewards. In reinforcement learning theory, the state value is discounted when the delivery of the reward is delayed, and Doya et al. suggested that 5-HT regulates this reward discounting rate (Doya, 2002; Tanaka et al., 2004). Indeed, the 5-HT level and firing rate in the DRN increased when rats waited to obtain rewards, and the level of neuronal firing was correlated with successful waiting (Miyazaki et al., 2010, 2011). Such “wait to obtain a reward” behavior might be originally initiated by the reward signal that activates the dopamine system, which then promotes behavioral vigor or activation, and at the same time, the subsequent activation of the DRN is necessary for the successful withholding of responses to obtain rewards.

## 5-HT and aversive information processing

The participation of 5-HT in aversive information processing has also been reported repeatedly. Strong evidence that 5-HT is involved in aversive information processing comes from the observation that there is a change in neuronal activity in the raphé nuclei or an increase in 5-HT levels in response to aversive stimuli. Stress-related stimuli activate immediate-early gene expression within the DRN (Pezzone et al., 1993). In the DRN of anesthetized rats, the majority of neurochemically identified 5-HT neurons with a clock-like firing pattern were phasically excited, whereas the majority of bursting 5-HT neurons were inhibited by noxious footshocks (Schweimer and Ungless, 2010). Activity level of the raphe nuclei is also modulated; it is increased under inescapable shocks (Grahn et al., 1999; Takase et al., 2004). Forced swimming induced an increase or decrease in 5-HT levels, as measured by microdialysis, depending on the brain region examined; its levels increased in the striatum, but decreased in the amygdala and lateral septum (Kirby et al., 1995).

The role of 5-HT in aversive information processing has multiple facets. First, several lines of evidence suggest that 5-HT modulates sensitivity to threat-related stimuli and punishment (for review, Deakin, 1991; Cools et al., 2008). A negative correlation between 5-HT levels and aversion has been demonstrated repeatedly, indicating the analgesic effect of 5-HT. Low levels of 5-HT in human subjects, achieved by acute tryptophan (the precursor of 5-HT) depletion, enhanced the responsiveness of several brain regions, especially the amygdala, to aversive stimuli, such as fearful faces and negative words (Hariri et al., 2002; Cools et al., 2005; Hariri and Holmes, 2006; Roiser et al., 2008). Low levels of 5-HT also alter the performance of a probabilistic reversal learning task by abnormally enhancing the impact of punishment, such as the inappropriate avoidance of less frequent punishment (Evers et al., 2005; Chamberlain et al., 2006). Note, however, that the role of 5-HT in a probabilistic reversal task may come from the changes in the processing of negative feedback signals *per se*, rather than changes in sensitivity to the error, because the changes in medial frontal activity did not differ between errors that were or were not followed by behavioral correction (Evers et al., 2005).

Just as decreased 5-HT function causes punishment processing to be enhanced, animal studies have shown that an increase in 5-HT levels inhibits responses to punishment. A well-known example is that increasing 5-HT levels via selective 5-HT reuptake inhibitors produces a potent reduction in the levels of anxiety, an effect underlying many anxiolytic drugs. Conditioned fear stress increases extracellular 5-HT levels in the rat medial prefrontal cortex, followed by a reduction of freezing behavior (Hashimoto et al., 1999). 5-HT also suppresses panic or defensive reactions (Maier and Watkins, 2005) and aggression (Marsh et al., 2002; Miczek et al., 2007). The DRN itself and the projection sites of 5-HT, such as the prefrontal cortex and amygdala, may be involved in this process (Graeff et al., 1996). The amygdala has an essential role in the learning and expression of conditioned fear to unconditional and conditional stimuli (Bechara et al., 1995; Ledoux, 2007), and the injection of the 5-HT reuptake blocker citalopram to the amygdala, which presumably enhances 5-HT levels, impairs fear conditioning (Inoue et al., 2004). Amygdala neurons that are excited by the electrical stimulation of glutamate-releasing inputs from the frontal cortex are inhibited by the concurrent iontophoresis of 5-HT, probably by the activation of GABA-releasing neurons through excitatory 5-HT receptors in the amygdala (Stutzmann et al., 1998; Stutzmann and Ledoux, 1999). Thus, deficient 5-HT function might result in the enhanced processing of harmful stimuli because of the diminished inhibitory modulation of excitatory sensory afferents, thereby enabling innocuous sensory signals to be processed by the amygdala as being emotionally salient.

Secondly, recent theoretical and experimental studies suggest that 5-HT does not operate solely as an affective (i.e., aversive) factor. Instead, the influence of 5-HT on aversive processing is evident on the junction of affective and activational factors; specifically, behavioral inhibition in the face of aversive predictions (Dayan and Huys, 2008, 2009; Boureau and Dayan, 2010). For example, in a task in which healthy human subjects decided to respond or not to obtain a reward or to avoid punishment, temporarily lowering 5-HT levels abolished the punishment-induced slowing of their response, but it did not affect the general inhibition of their motor response or sensitivity to aversive outcomes (Crockett et al., 2009). However, aversive predictions can be an instrumental process that links stimuli, responses, and outcomes, or they can be a Pavlovian process that links stimuli and outcomes. Here, further study revealed that 5-HT is involved in reflexive, Pavlovian aversive predictions because the latencies for the punished and non-punished responses were prolonged in the presence of punishment stimuli under acute tryptophan depletion (Crockett et al., 2012).

The third aspect of 5-HT-dependent neuronal processes associated with aversive experiences is behavioral control over a stressor. Generally, the emotional consequences related to aversive events are less severe if the subjects have control over the aversive events, and a lack of control of stress leads to mood and anxiety disorders. Experimentally, animals exposed to inescapable stressors subsequently exhibit “learned helplessness,” a set of behavioral changes that include an impaired ability to escape from aversive events, increased fear conditioning and anxiety, a potentiated response to addictive drugs, and altered pain sensitivity. It has been suggested that 5-HT is involved in this “reduction of action” after a stressful, uncontrollable situation. Indeed, the activity of DRN 5-HT neurons, as measured by Fos expression (Grahn et al., 1999), and 5-HT levels, as measured by *in vivo* microdialyzis (Maswood et al., 1998), in the DRN or its projection sites (Amat et al., 1998; Bland et al., 2003a,b) were enhanced under an inescapable stress, such as tailshock, but not under an escapable stress. Further, the intense activation of DRN 5-HT neurons by an uncontrollable stress sensitizes these neurons for a period of time (Amat et al., 1998). The inactivation of 5-HT blocks the occurrence of these behavioral changes (Maier et al., 1994, 1995).

One possible mechanism for the activation of the DRN under inescapable stress is input to the DRN from the habenula. Lesions of the habenula severely attenuate the rise in 5-HT levels in the DRN under both escapable and inescapable stress, thus eliminating the difference between them and producing behavioral indifference (Amat et al., 2001). The frontal cortex may also be involved in this process. When a stressor is controllable, the DRN is no longer activated by the stressor due to inhibitory signals from the ventral medial prefrontal cortex (Amat et al., 2005). The role of the ventral medial prefrontal cortex may be to detect the fact that the stressor is controllable rather than to escape learning *per se*. If controllable, the prefrontal cortex inhibits DRN activation and thus prevents learned helplessness. A recent study used an optogenetic approach to reveal more detailed neuronal circuits that support such behavioral changes; activation of the prefrontal-DRN pathway is causally involved in an increase in effortful movement during the forced swim test, which is a challenging and inescapable situation, whereas activation of the prefrontal-habenula pathway caused the opposite effect (Warden et al., 2012). Note that such a situation or state-dependence is also documented for single neuronal activity. For example, DRN neurons in the rat responded to a tone differently, depending on the reward and no-reward context (Li et al., 2013).

Fourth, an increase of 5-HT may regulate the processing of stress via the activation of pituitary and adrenal functions (Vernikos-Danellis et al., 1977), which have bi-directional interactions with the 5-HT system. There is a dense projection of corticotropin-releasing factor (CRF) neurons to the raphé nuclei in rats (Cummings et al., 1983; Lowry et al., 2008) and humans (Austin et al., 1997). A subpopulation of CRF-containing neurons is present in the dorsomedial part of the DRN, and dual-labeling immunohistochemistry revealed that almost all CRF-containing neurons are serotonergic (Commons et al., 2003). Intracerebroventricular injections of the selective CRF2 receptor agonist urocortin 2 increased the activity of serotonergic neurons (Abrams et al., 2004; Staub et al., 2005, 2006). However, the effects of CRF on the DRN appeared to be either excitatory or inhibitory, probably depending on the location of the recorded neurons within the DRN, e.g., neurons in the ventromedial region were inhibited, whereas neurons in the dorsomedial and lateral wings had variable responses (Kirby et al., 2000). In addition, CRF-containing axons from the dorsomedial DRN project to CRF-containing neurons of the central nucleus of the amygdala, a stress related area and a part of the central autonomic system (Petrov et al., 1994). There is also a dense projection of 5-HT neurons to the suprachiasmatic nucleus, which in turn regulates the secretion of CRF from the hypothalamus and, consequently, adrenocorticotropic hormone (ACTH) release. Thus, it is important to emphasize the two roles of 5-HT in the mammalian brain, i.e., as a neurotransmitter and a hormonal factor. These two aspects may be related to each other as a recent study showed that the negative prediction error signal in the ventral striatum is strengthened under stress (Robinson et al., 2013).

## Single unit recordings from the DRN

The anatomical and pharmacological evidence reviewed above suggests that 5-HT has potent effects on reward and punishment, and that its effects are tightly regulated by the neural circuitry interacting with the DRN. A missing piece of this puzzle is precisely how DRN neurons behave while reward-oriented behavior unfolds in real time. The studies reviewed above typically manipulated DRN function over long timescales, such as hours, and over a wide spatial extent, altering 5-HT function in multiple brain regions simultaneously. Yet the reward-related processes that the DRN regulates, including seeking, consuming, and learning about rewards, are performed during natural behavior within the span of minutes or seconds. In addition, while the behavioral-pharmacological experiments examined how 5-HT is utilized at the projection sites, much less is understood about in which situations DRN neurons secrete 5-HT.

**Figure 2:**
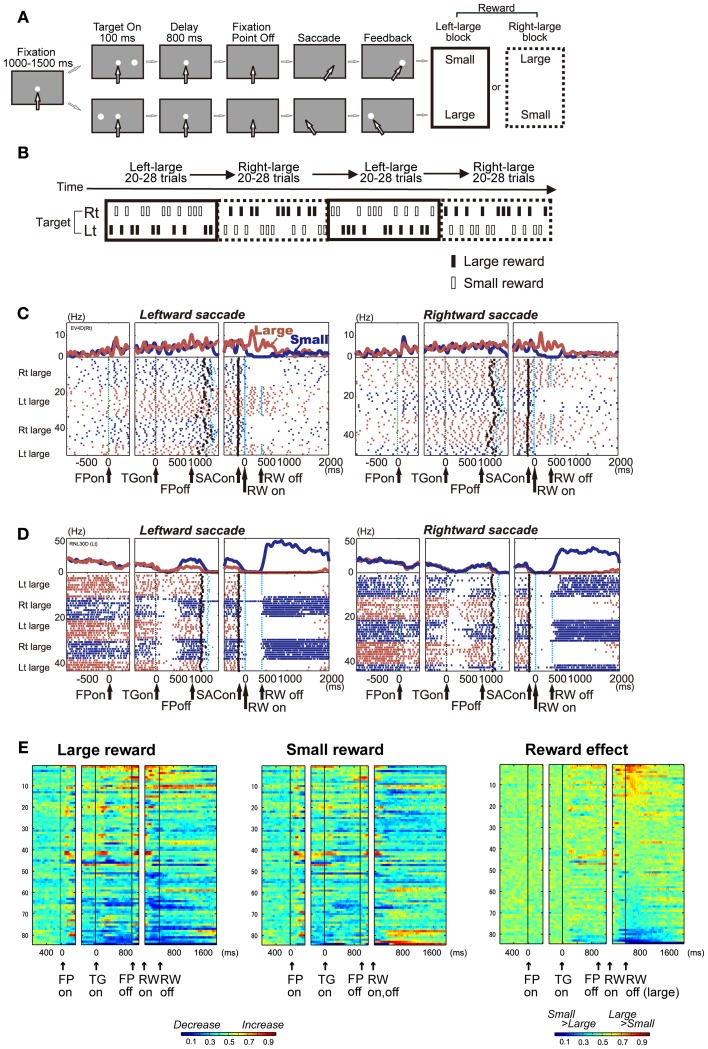
**(A)** One direction rewarded memory guided saccade (1DR-MGS) task. After the monkey fixated on the central fixation point for 1200 ms, one of the two target positions was flashed for 100 ms. After the fixation point disappeared, the monkey made a saccade to the cued position to receive a liquid reward. The white arrows indicate the direction of gaze. In a block of 20–28 trials (e.g., left-large block), one target position (e.g., left) was associated with a large reward and the other position (e.g., right) was associated with a small reward. The position-reward contingency was then reversed (e.g., right-large block). **(B)** Left-large and right-large conditions were alternated between blocks with no external cue. The location of the target was determined pseudo-randomly. **(C,D)** Examples of the activity of two DRN neurons in the 1DR-MGS task. The activity in the large- and small-reward trials is shown in red and blue, respectively. The histograms and raster plots are shown in three sections: the left section is aligned to the time of fixation point onset (FPon), the middle section is aligned to target onset (TGon) and fixation point offset (FPoff), and the right section is aligned to reward onset (RWon). The black dots indicate saccade onset (SACon); the blue dots indicate reward onset and offset. Note that reward offset (RWoff) applies only to the large-reward trials. **(E)** Population activity of DRN neurons in the 1DR-MGS task (*n* = 84). The activity of each neuron is presented as a row of pixels. Left and center: changes in the neuronal firing rate from baseline are compared in the large- and small-reward trials. The color of each pixel indicates the ROC value based on the comparison of the firing rate between a control period just before fixation onset (400 ms duration) and a test window centered on the pixel (100 ms duration). This analysis was repeated by moving the test window in 20-ms steps. The warm colors (ROC > 0.5) indicate increases in the firing rate relative to the control period, while the cool colors (ROC < 0.5) indicate decreases in the firing rate. Right: changes in reward-dependent modulation. The ROC value of each pixel was based on the comparison of the firing rate between the large- and small-reward trials. The warm colors (ROC > 0.5) indicate higher firing rates in the large-reward trials than in the small-reward trials. Modified from (Nakamura et al., 2008).

To understand which aspects of cognitive behavior are encoded by the activity of DRN neurons in real time, several research groups have measured the activity of single DRN neurons while animals performed behavioral tasks. In the following section, I will introduce our studies in primates performing “biased reward saccade tasks” (Figures 2A,B) (Bromberg-Martin et al., 2010). Using saccades as a behavioral measure is advantageous for several reasons. First, the measurement and assessment of changes in behavior, i.e., eye movement, are relatively simple. Second, the neuronal circuit for the generation of eye movements is well established.

While the activity of DRN neurons was found to be correlated with a variety of events, including movements, stimulus identity, and response direction (Ranade and Mainen, 2009), we found that reward information is one of the most influential factors for the modulation of DRN neuronal activity. A comparison of DRN activity with that of midbrain dopamine neurons also highlighted the distinct aspects of reward coding by different monoamine neurotransmitters.

### Single neuronal activity of the primate DRN in a biased reward saccade task

Nakamura et al. recorded DRN neuronal activity while monkeys performed memory-guided saccade tasks with a biased reward schedule (Nakamura et al., 2008). After fixation on a central fixation point, a target flashed briefly to either the left or right. After a delay of 800 ms, the animal made a saccade in the direction where the target was previously presented (Figure 2A). The main feature of the task was the block design of the reward schedule (Figure 2B). For every 20–28 consecutive trials, called a block, one direction was always associated with a large reward, while the other direction was always associated with a small reward (e.g., right-large, left-small). Thus, we can measure the effect of the expectation and receipt of a certain reward size on neuronal activity. In addition, this target location-reward size contingency was switched between blocks (e.g., right-large, left-small to right-small, left-large) without an explicit signal, which caused the receipt of an unexpectedly large or small reward on the very first trial of each block. This feature enabled us to measure the effect of the positive and negative reward prediction error.

### DRN neurons encode the expected and received reward value

We found that many DRN neurons exhibited task-related activity that was modulated by the expected and received reward value. Figure 2C shows a representative example. This neuron exhibited an increase in activity after the onset of the fixation point (FPon) followed by regular and tonic firing until reward onset (RWon). The activity further increased after the onset of a large reward, but ceased after the onset of a small reward, and this trend lasted tonically after reward onset. Another example neuron in Figure 2D showed an opposite modulation pattern. This neuron exhibited a decrease in activity after the onset of the fixation point followed by a tonic increase for small reward trials and suppression for large reward trials.

Reward-dependent modulation in activity was commonly observed in the population of DRN neurons. Figure 2E illustrates the time course of activity modulation using receiver operating characteristic (ROC) analysis by comparing the firing rate of each neuron for large (Figure 2E, left) and small (Figure 2E, middle) reward conditions to their baseline activity during 400 ms before fixation onset. During both periods before and after reward delivery, called the pre- and post-reward periods, respectively, many DRN neurons exhibited tonic increases (shown in warm colors) or decreases (cool colors) in activity. Figure 2E, right, compares the activity of each neuron between the large- and small-reward trials. The tonic reward effect was present in many neurons during both the pre- and post-reward periods.

There was a notable difference in reward-dependent modulation between the pre- and post-reward periods, indicating a different source of information. For each neuron, the change in activity during the pre-reward period, compared with baseline activity, tended to be in the same direction in both the large- and small-reward trials. On the contrary, the change in activity during the post-reward period, compared with baseline activity, tended to be in the opposite direction. For example, for the neuron shown in Figure 2A, the pre-reward activity increased compared with the baseline in both the large- and small-reward trials. On the other hand, its post-reward activity increased in the large-reward trials, but was inhibited in the small-reward trials relative to its baseline activity before fixation point onset. Thus, the main cause of the reward effect during the pre-reward period was that the change in activity tended to be stronger in the large-reward trials than in the small-reward trials. Conversely, the reward-dependent modulation of post-reward activity was caused by the modulation of activity in the opposite direction, depending on the reward value.

### DRN neurons keep track of the expected and received reward value

DRN neurons exhibited a tonic increase or decrease in activity that was modulated by the expected and received reward value. What do these tonic changes encode? One possibility is that this tonic modulation of activity encodes sustained aspects of motivated behavior, such as the state of expectation of future rewards for each moment. If so, the activity during the fixation period may represent the expected value of the performance of the task itself. This is because the animal did not know the exact value of the upcoming reward during the fixation period, but knew the averaged expected reward value, which should be a value between the large and small rewards. After target presentation, the exact expected/received reward value was known. If the neurons encoded the behavioral tasks primarily in terms of their reward value throughout a trial, then the neurons excited during the fixation period should be preferentially excited by the reward cues (i.e., carrying positive reward signals), whereas the neurons inhibited during the fixation period should be preferentially inhibited by the reward cues (i.e., carrying negative reward signals). Conversely, if the neurons encoded the fixation period and reward value in an independent manner, then there should be no systematic relationship between fixation- and reward-related activity.

**Figure 3:**
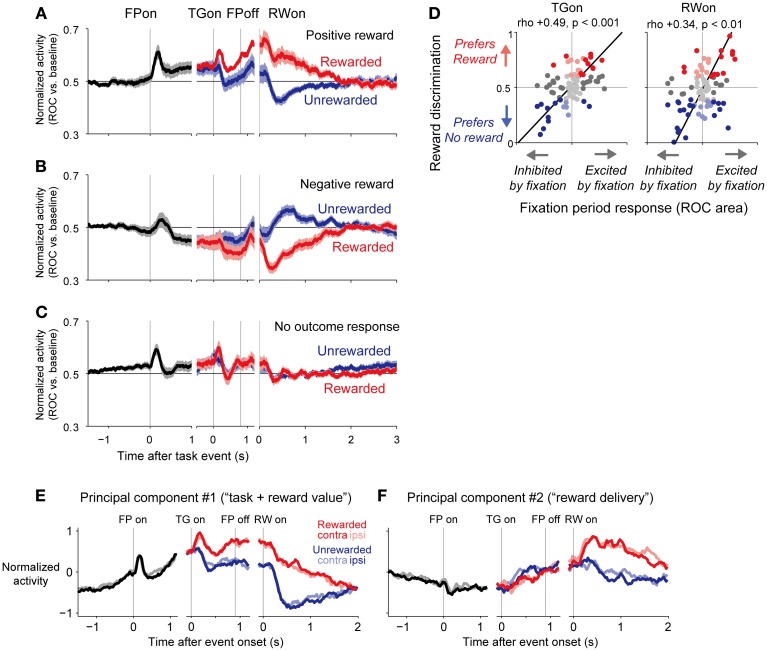
**Population average activity of dorsal raphé neurons separated by their reward signals in response to the outcome. (A–C)**, Normalized activity is shown for the 1DR-MGS task, separately for positive-reward cells **(A)**, negative-reward cells **(B)**, and non-outcome responsive cells **(C)** The neurons were sorted into these categories based on significant reward discrimination during a 150–450-ms window after outcome onset (gray bar on the x-axis; *p* < 0.05, Wilcoxon rank-sum test). Thick lines, mean normalized activity; light shaded areas, 1 SEM. **(D)** Neural activity during the fixation period was positively correlated with reward coding during the target and outcome periods. The x-axis indicates the fixation period response, which was measured as the ROC area for each neuron for discriminating between its firing rate at 500–900 ms after fixation point onset vs. the pre-fixation period at 0–400 ms before fixation point onset. The y-axis indicates reward discrimination, which was the difference in reward responses between the large- and small-reward trials. The text indicates rank correlation (rho) and its *p*-value. The dark dots indicate neurons with a significant excitation or inhibition during the fixation period. The colored dots indicate neurons with significantly higher activity during the rewarded trials (red) or during the unrewarded trials (blue) (*p* < 0.05, Mann-Whitney *U*-test). The black lines indicate the line of best fit calculated using type 2 least-squares regression. **(E,F)** The first **(E)** and second **(F)** principal components of dorsal raphé neural activity profiles during the memory-guided saccade. Curves represent the normalized firing rate of the principal component during the fixation period (black) and after the onset of the rewarded (red) and unrewarded (blue) target, separately for the contralateral-rewarded block (dark colors) and ipsilateral-rewarded block (light colors). The first principal component indicated tonically increased activity during the fixation period and positive-reward coding during the target, memory, and outcome periods. The second component indicated tonically increased activity in response to reward delivery. Modified from (Bromberg-Martin et al., 2010).

Analysis revealed that there was indeed a strong correlation between the tonic activity level of a neuron during the fixation period and its encoding of reward-related cues and outcomes. For example, neurons like the one presented in Figure 2C showed a sustained elevation in activity during the fixation period. After reward delivery, these neurons responded with a positive reward signal, with higher activity in response to the large- than to the small-reward trials. Other neurons, like the one presented in Figure 2D, showed a sustained suppression in activity during the fixation period, with higher activity in unrewarded trials than in rewarded trials. The population average of normalized activity was computed separately for neurons with positive, negative, or no significant reward signals in response to the outcome (Figures 3A–C). Neurons with positive reward signals for the outcome had elevated activity during the early period of the task (Figure 3A); if the rewarded target appeared, their activity was elevated further, whereas if the unrewarded target appeared, they returned to near baseline. Neurons with negative reward signals had suppressed activity during the early period of the task (Figure 3B); if the rewarded target appeared, their activity was suppressed further, whereas if the unrewarded target appeared, they returned to near baseline. Neurons with no significant reward signals had a tendency for small phasic responses to the fixation point and targets and slightly elevated activity during the task (Figure 3C).

The activity of a neuron during the fixation period was strongly positively correlated with its degree of reward discrimination during the post-target and post-reward periods (Figure 3D). If the elevation of activity during the fixation period was stronger, the neuron had higher discrimination of a positive-reward signal; with stronger activity for large- than small-reward trials during the post-target and post-reward periods. If the inhibition of fixation activity was stronger, the neuron had higher discrimination of a negative-reward signal; with stronger activity for small- than large-reward trials during the post-target and post-reward periods. Thus, most DRN neurons responded to the initiation of a behavioral task in the same direction as they responded to the reward cues and outcomes, and those neurons with stronger task coding also had stronger reward coding. These two signals combined so that the level of DRN activity tracked progress throughout the task toward obtaining future rewards. This form of correlated task and reward coding had a dominant influence on DRN neurons, and it was not simply one of many systematic forms of task and reward encoding.

So far, the analysis showed that DRN neurons encoded the information of the task (i.e., fixation period activity) and reward outcome in a correlated manner. However, it did not analyze whether this correlation had a dominant tendency or it was merely one of many systematic forms of task and reward encoding. The analysis was also performed only for restricted periods of the task, which were determined tentatively. To characterize the activity patterns of neurons during all task phases in an unbiased manner, we applied principal component analysis (Richmond and Optican, 1987; Paz et al., 2005). In this analysis, the activity of each neuron is described as a linear combination of the major components of activity that varied systematically with the task variables; the first principal component represents the most common pattern of neural activity with the greatest amount of variance, the second principal component explains the second most common pattern of neural activity, and so on. Then, the activity profile of every neuron may be reconstructed as the sum of its mean neural activity profile plus a weighted combination of the principal components. If a neuron is assigned a component positive weight, then its activity is positively related to the time series of that component. Conversely, if a neuron is assigned a component negative weight, then its activity is negatively related to the time series of that component.

In the DRN population of neurons, the first principal component (Figure 3E) indicated a positive correlation between task onset-related activity and reward coding. It consisted of a gradual increase in tonic activity during the inter-trial interval and after fixation point onset, followed by an additional increase in tonic activity in response to the rewarded target. The second principal component (Figure 3F) had a prolonged tonic change in activity after a reward was delivered. Thus, whereas the first component resembled “task-reward value coding,” the second component resembled “reward delivery coding.”

Note that principal component analysis treats neural activity as a linear combination of orthogonal components; if the “true” components underlying neural activity are combined non-linearly or are not orthogonal, the principal components may not represent them perfectly. Nevertheless, further analysis indicated that only the first two principal components explained significantly more variance in activity than would be expected under the null hypothesis that there were no systematic patterns in the data using shuffled datasets (Bromberg-Martin et al., 2010). Thus, these principal components explained most of the systematic variation in neuronal activity that was related to task events.

### Difference from dopamine neurons

**Figure 4:**
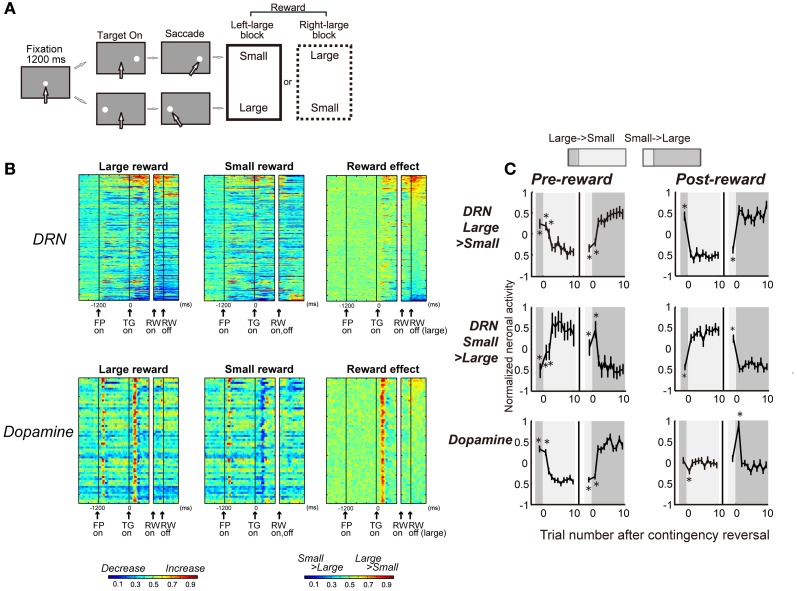
**DRN neurons and dopamine neurons encode different aspects of the reward. (A)** Visually guided version of the one direction rewarded saccade (1DR-VGS) task. **(B)** Activity of 167 DRN neurons and 64 dopamine neurons for the 1DR-VGS task. The same format is used as in Figure 2E. **(C)** Changes in neuronal activity with the reversal of position-reward contingency. Top and middle: DRN neurons with large- and small-reward preferences, respectively. Bottom: dopamine neurons. For each group, the activity during the pre-reward period (400 ms after target onset) is shown on the left, and the activity during the post-reward period (400–800 ms after reward onset for DRN neurons; 0–400 ms after reward onset for dopamine neurons) is shown on the right. For each graph, the left panel shows large-to-small reward reversal; the right panel shows small-to-large reward reversal. The large-reward trials are indicated by dark gray; the small-reward trials are indicated by clear areas (as in the top). Shown are the mean and SE of the normalized neuronal activity for the n-th trial after contingency reversal. The asterisks (^*^) indicate activity that was significantly different from the activity in the last five trials of the block with the reversed contingency (*p* < 0.01, Mann-Whitney *U*-test). Modified from (Nakamura et al., 2008).

Reward-dependent modulations of the activity of DRN neurons were distinctively different from those observed in putative dopamine neurons for the same task (the visually guided version of the biased-reward saccade task, Figure 4A). First, whereas DRN neurons responded to both the reward-predicting stimulus and the reward itself (TGon and RWon, respectively, Figure 4B), dopamine neurons predominantly responded to the reward-predicting sensory stimulus (TGon). Second, whereas the DRN contains neurons that preferred larger rewards and neurons that preferred smaller rewards, dopamine neurons invariably preferred larger rewards (i.e., are excited by larger rewards). Third, whereas DRN neurons reliably coded the value of the received reward, whether or not it was expected, dopamine neurons responded to a reward only when it was larger or smaller than expected. Figure 4C shows the changes in neuronal activity during the pre- and post-reward periods when the target location-reward value contingency was switched. The activity of positive and negative reward-coding DRN neurons exhibited the expected (pre-reward) and received (post-reward) reward values. The changes in the activity of dopamine neurons during the pre-reward period were similar to those of DRN neurons. However, unlike DRN neurons, dopamine neurons responded to reward delivery only when the cue position-reward contingency was switched so that the reward was unexpectedly small or large, consistent with the prediction error hypothesis (Schultz, 1998; Kawagoe et al., 2004). Finally, whereas DRN neurons typically exhibited tonic responses, dopamine neurons exhibited phasic responses. Thus, DRN neurons provide tonic signals related to the expected and received reward values, unlike dopamine neurons that provide phasic signals related to the reward prediction error.

## Discussion

The characteristic features of the activity of DRN neurons observed in the biased-reward saccade tasks were a tonic response pattern and stronger modulation for the most valuable option in either a positive or negative manner. The tonic activity underlying the expected reward value indicates its role in subjective motivation to obtain a reward or “wanting;” the response to the received reward value indicates its role in a subjective hedonic experience or “liking” (Berridge and Kringelbach, 2008). Correlated fixation-period activity, which represents the task value, and post-outcome activity, which represents the value of the received reward, indicate that DRN activity encodes behavioral tasks primarily in terms of their reward value throughout a trial. The principal components, which explained the majority of activity patterns, indicate that this reward coding, aside from other possible sensory-motor coding, is the major component of DRN activity. Conversely, DRN neurons do not appear to encode the prediction error signal of appetitive or aversive events.

**Figure 5:**
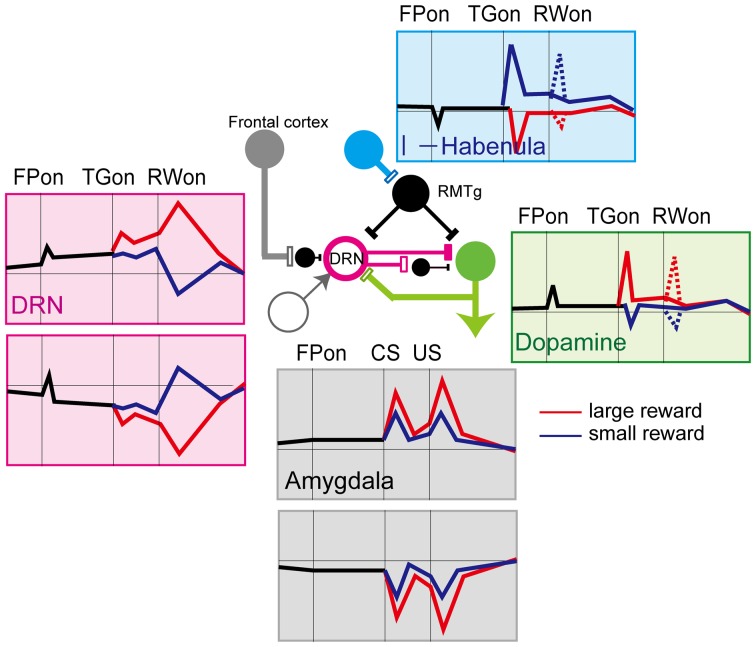
**Schematics of the activity of neurons in the different brain regions that project to the DRN.** Red lines, large reward, blue lines, small reward. Activity for the biased-reward saccade task in dopamine (Kawagoe et al., 2004), lateral habenula (Matsumoto and Hikosaka, 2007, 2009), and DRN neurons (Nakamura et al., 2008), and for the Pavlovian conditioning task in the amygdala (Belova et al., 2007, 2008) is shown.

Possible sources of the pre-reward activity (i.e., the response to fixation and target) may be dopamine neurons in the SNc, VTA, and lateral habenula (Figure 5). Since dopamine neurons are excited by a large reward-predicting cue, DRN neurons would also be excited by the same cue (Kawagoe et al., 2004). Indeed, during the pre-reward period, a large-reward preference was more common (~20% of all task-related DRN neurons) than a small-reward preference (~5%). The main projection from the lateral habenula to the DRN is, on the other hand, inhibitory. Using the same biased-reward saccade tasks, Matsumoto et al. showed that lateral habenula neurons were excited by stimuli that predict small rewards and were inhibited by a large-reward predicting cue (Matsumoto and Hikosaka, 2007). Such modulation of habenula activity would then be inversely translated into the large-reward preference of DRN neurons via inhibitory neurons in the RMTg.

The post-reward responses of DRN neurons are unlikely to be derived from dopamine or habenula neurons because neither of them exhibit post-reward responses, except on the first trial after the block was switched. Possible origins of the post-reward activity include the amygdala, hypothalamus, and medial prefrontal cortex. In the post-reward period, unlike the pre-reward period, the direction of modulation relative to the baseline was often opposite between the large- and small-reward trials. This observation indicates different sources of activity for the large- and small-reward trials. It was also found that one population of DRN neurons showed a large-reward preference and another population showed a small-reward preference. One possible interpretation would be that the source of the two kinds of reward-related signals (small > large and large > small) are represented in other brain areas, such as the anterior cingulate cortex (Niki and Watanabe, 1979; Amiez et al., 2006), and these signals are transmitted to the DRN (Arnsten and Goldman-Rakic, 1984). Another possible source is the amygdala. Amygdala neurons, like DRN neurons, tracked progress throughout a behavioral task, such that the response of a neuron to the start of the task was strongly correlated with its response to the reward cue and outcome. They also include both positive and negative coding neurons (Belova et al., 2008). Another possibility is that the reward information originated from the same group of neurons, but was transmitted to the DRN by different mechanisms; one directly, the other indirectly, via inhibitory connections. For example, the ventral medial prefrontal cortex inhibits 5-HT neurons in the DRN by targeting local GABAergic interneurons (Varga et al., 2001). Such multi-channeled inputs would enable the DRN to integrate positive and negative reward values independently over time.

## Possible functions of the DRN in reward processing and the direction of future research

The tonic activity of DRN neurons may be ideal to signal a continuous level of motivation and hedonic experience throughout the performance of a task. Such a signal may provide a “reward context” signal to the targets of DRN projections, where the signal may be used differently depending on the type of 5-HT receptor present.

First, the sustained reward signals in the DRN could be used to track the value of the current behavioral state. Such estimated values have an important role in theories of reinforcement learning, which suggest that the prediction error signal of dopamine neurons is calculated as the difference between the actual and expected reward values. Thus, DRN activity could contribute to the computation of prediction errors by providing the current state of the expected reward value.

Second, DRN activity may report the long-term averaged reward, rather than immediate, phasic reward information (Daw et al., 2002). In real life, one needs to integrate flows of information, including both appetitive and aversive events and situations, to achieve better decision making to adapt to external changes. The tonic activation patterns of DRN neurons may be useful in integrating appetitive and aversive information coming from different sources (as in Figure 1, left) over a substantial period of time.

The activity of DRN neurons observed in behaving monkeys is characterized by a mirror-image pattern of reward coding by different subsets of neurons, namely, positive and negative reward coding (Figure 5). The current theoretical account of 5-HT function is that it may be involved in behavioral inhibition in the face of punishment (Cools et al., 2011). Thus, the neuronal activity data in the DRN of behaving animals appears partially unexpected because some neurons showed stronger activity in the expectation of large rewards. This seemingly inconsistent finding may be because different groups of neurons might map onto neurochemically or anatomically different subgroups. In the neurochemical account, it is possible that the negative coding DRN neurons could be serotonergic projection neurons, while the positive coding ones may be GABAergic interneurons. Clarifying the underlying cell properties is essential for further understanding of the function of 5-HT (Schweimer and Ungless, 2010). In the anatomical account, neurons may respond differently depending on the circuit in which they are involved. For example, a recent single unit recording study in primates (Inaba et al., 2013) reported that neurons that prefer rewards tend to be distributed more rostrally, while neurons that prefer no rewards were distributed more caudally. It is possible that these different types of neurons may be involved in different anatomical circuits in the brain.

The mirror-image activity of different sets of DRN neurons also suggests that their function may be highly context-dependent. The DRN is anatomically and functionally linked to different circuits involving different brain structures, such as the frontal cortex, amygdala, basal ganglia, and dopamine neurons, and context here may depend on which circuit is mainly involved. Indeed, Warden et al. showed that stimulation of specific projections from the medial frontal cortex to the DRN caused changes in animals' movement in a challenging situation (the forced swim test), while stimulation of the overall DRN caused, in addition to the usual effects observed in a challenging situation, a general increase in movement (the open field test) (Warden et al., 2012). The activation of a specific pathway of DRN neurons with specific task-related activity may support the context-dependent selection of value-based decision making.

Another possible function of the seemingly opposite signals might be the interaction between the 5-HT and other systems, including dopamine systems, to compute appetitive and aversive information in a balanced manner (Figure 5). As in Solomon and Corbit's affective dynamics model (Solomon and Corbit, 1974), the value of rewards is treated as a continuous signal rather than the pulsatile pattern of the value signal, and the tonic DRN activity we observed may correspond to this signal. With the normal level of DRN activity and 5-HT, the baseline activity of dopamine neurons may be tonically suppressed. In addition, phasic appetitive and aversive event-indicating cues would drive both dopamine and DRN neurons which inhibit, at least partly, dopamine neurons, simultaneously. Thus, the DRN would attenuate the strength of responses of the dopamine system to appetitive and aversive events. This process might have the advantage of maintaining equilibrium in terms of reward to prevent excessive positive or negative value coding. Given the variety of 5-HT receptors and their functions, this scheme is, of course, simplistic. It should also be clarified whether the regulation of the reward circuit by 5-HT is always dopamine-dependent, like the proposed scheme, or it can act independently and directly. Combined research of circuit-specific manipulation such as the optogenetic approach and detailed analyses of neural activity in relation to changes in behavior would lead to a clear understanding of the role of 5-HT.

### Conflict of interest statement

The author declares that the research was conducted in the absence of any commercial or financial relationships that could be construed as a potential conflict of interest.
